# Reactive Oxygen Species as Additional Determinants for Cytotoxicity of *Clostridium difficile* Toxins A and B

**DOI:** 10.3390/toxins8010025

**Published:** 2016-01-18

**Authors:** Claudia Frädrich, Lara-Antonia Beer, Ralf Gerhard

**Affiliations:** 1 Postgraduate Course for Toxicology and Environmental Toxicology, Institute for Legal Medicine, University of Leipzig, Johannisallee 28, Leipzig 04103, Germany; fraedrich_claudia@hotmail.de; 2 Institute of Toxicology, Hannover Medical School, Carl-Neuberg-Str. 1, Hannover 30625, Germany; beer.lara-antonia@mh-hannover.de

**Keywords:** *Clostridium difficile* infection, reactive oxygen species, cytotoxicity, NADPH oxidase, neutrophils, Rho GTPases, toxin

## Abstract

*Clostridium difficile* infections can induce mild to severe diarrhoea and the often associated characteristic pseudomembranous colitis. Two protein toxins, the large glucosyltransferases TcdA and TcdB, are the main pathogenicity factors that can induce all clinical symptoms in animal models. The classical molecular mode of action of these homologous toxins is the inhibition of Rho GTPases by mono-glucosylation. Rho-inhibition leads to breakdown of the actin cytoskeleton, induces stress-activated and pro-inflammatory signaling and eventually results in apoptosis of the affected cells. An increasing number of reports, however, have documented further qualities of TcdA and TcdB, including the production of reactive oxygen species (ROS) by target cells. This review summarizes observations dealing with the production of ROS induced by TcdA and TcdB, dissects pathways that contribute to this phenomenon and speculates about ROS in mediating pathogenesis. In conclusion, ROS have to be considered as a discrete, glucosyltransferase-independent quality of at least TcdB, triggered by different mechanisms.

## 1. Introduction

*C. difficile* is a gram-positive anaerobic bacterium that causes antibiotic-associated diarrhoea and colitis. The intestinal disease ranges from asymptomatic colonization to mild diarrhoea and more severe disease syndromes including pseudomembranous colitis or in rare cases toxic megacolon [1]. In 2015 the Centers for Disease Control and Prevention estimated more than 450,000 cases a year with almost 30,000 associated deaths.

The main virulence factors of *C. difficile* are two members of the large clostridial cytotoxin family, toxin A (TcdA) and toxin B (TcdB). TcdA and TcdB are large (308 kDa and 270 kDa, respectively) glucosyltransferases that irreversibly inactivate Rho and Ras family GTPases, including Rho, Rac, Ras, Ral and Cdc42, within the cell [2,3,4]. Both toxins are responsible for massive fluid secretion, colonic tissue necrosis and inflammation associated with disease [5]. In addition to the major toxins, *C. difficile* produces a number of other putative virulence factors, e.g., the CDT binary toxin, which is produced in approximately 30% of all hypervirulent strains [6,7,8]. The genes encoding toxins A and B (tcdA and tcdB, respectively) are on the 19.6-kb region of the chromosome, so-called pathogenicity locus or PaLoc, along with two regulatory genes (tcdC and tcdR) and a gene (tcdE) encoding a protein proposed to function as a porin facilitating the release of toxins A and B through permeabilization of the cell wall [9,10], although its essential role in the release of the toxin is still under discussion [11,12].

TcdA and TcdB are homologous AB-structure toxins and either toxin can be divided into two components: an enzymatic A-subunit and a B-subunit, which is involved in delivery of the A-subunit into the cytosol of the host cell. The *N*-terminal A-subunit is a glucosyltransferase domain (GTD) that inactivates host GTPases by mono-glucosylation. The B subunit comprises four domains, of which three are characterized: the very *C*-terminal combined repetitive oligopeptides (CROPs) serve as one receptor binding domain by binding to carbohydrate structures [13,14]. A central hydrophobic region including the pore-forming domain is essential for membrane insertion and builds a hatch for translocation of the GTD into the cytosol, and the cysteine protease domain which releases the GTD by autoprocessing. The fourth domain residing between the hydrophobic region and the CROPs is as yet poorly characterized in its function but takes part in binding of further receptor(s) [15]. The chondroitin sulfate proteoglycan 4 (CSPG4) and poliovirus receptor like protein 3 (PVRL3) were identified as receptors for TcdB [16,17] and sucrose-isomaltase and p-glycoprotein have been reported to serve as receptors for TcdA [18,19]. The mechanism of intake of TcdA and TcdB can be divided into four consecutive steps: (1) binding/internalization step; (2) pore-formation and translocation of the GTD across the membrane; (3) release of GTD by autoproteolysis and (4) inactivation of host GTPases by glucosylation [20]. Following receptor binding at the host cell, the toxins are endocytosed via clathrin-dependent and -independent pathways [21,22]. After endocytosis, the *N*-terminal GTD is translocated through the early endosomal membrane [23]. Translocation into the host cell cytosol requires acidification of the endosome by vesicular H^+^-ATPase. In 2000, Qa’Dan and co-workers showed that bafilomycin A1, a potent inhibitor of the endosomal vacuolar ATPase, inhibits the toxin B-mediated cytotoxicity [24]. The low pH of endosomes induces conformational changes of the toxins by which the hydrophobic region of the protein toxin is exposed and enables membrane insertion. This membrane insertion is associated with pore formation [24,25,26]. Following pore formation, binding of inositol hexakisphosphate activates the autocatalytic cleavage between the GTD and the cysteine protease domain, with the subsequent release of the active *N*-terminal GTD into the cytosol [27].

This activated *N*-terminal domain which is released into the cell inactivates small GTPases. Rho GTPases play an important role in many cellular processes like organisation of the actin cytoskeleton, cell-cell adhesion, cytokinesis and secretion. They are controlling epithelial barrier function and are involved in the signaling and motility of immune cells [23]. Glucosylation of Rho GTPases like RhoA, Rac1 and Cdc42 results in the disruption of cell-cell junctions and causes reorganization of the actin cytoskeleton. Inactivation of Rho GTPases induces a stress activated pro-inflammatory response such as up-regulation of interleukin-8, interleukin-1β (IL-1β) or tumor necrosis factor-α (TNF-α) [28,29]. Both toxins cause a cytopathic effect in cells, which is complete rounding of cells due to inhibition of Rho GTPases and reorganization of the actin cytoskeleton [30]. Compared to TcdA, TcdB is about 1000 times more potent in inducing cell rounding in most cell lines [31,32]. The loss of structural integrity and “cell rounding” are in general associated with subsequent apoptosis via caspase-8 and -9 (initiator caspases) and caspase-3 (executor) dependent pathways [33,34,35].

While programmed cell death, *i.e*. caspase-dependent apoptosis, is a delayed consequence of inhibition of Rho GTPases, an increasing number of publications report about an early necrotic or non-apoptotic cell death specifically induced by TcdB [34,36,37]. This review will focus on the newly discovered mechanism of cell death and the relevance of ROS production in TcdB-treated cells.

## 2. Increased ROS Triggered by TcdA and TcdB

### 2.1. ROS Contribute to Inflammation, Cell Death and Redox Signaling

ROS are involved in many diseases and its role in cell death has been reported in lots of literature. ROS is a collective term that includes oxygen radicals, such as superoxide (O_2_^•−^), hydroxyl (HO^•^), peroxyl (RO_2_^•^) and alkoxyl (RO^•^) radicals and non-radical oxidizing agents, such as hydrogen peroxide (H_2_O_2_) and hypochlorous acid (HOCl) [38]. Other reactive species are nitrogen species such as nitric oxide (NO^•^), nitric dioxide (NO_2_^•^) and peroxynitrite (OONO^−^) [39].

ROS are normal by-products of many cellular metabolism and involved in enzymatic reactions, mitochondrial electron transport, signal transduction, activation of nuclear transcription factors, gene expression and the antimicrobial action of neutrophils and macrophages. In mammalian cells, a variety of enzymatic and non-enzymatic processes generates ROS. The main sources are enzymatic reactions catalyzed by nicotinamide adenine dinucleotide phosphate oxidase (NADPH oxidase), xanthine oxidoreductase (XOR) and myeloperoxidase (MPO) [40]. Overproduction of ROS and their derivatives can cause oxidative DNA and protein damage, which may play an important role in the initiation and progression of carcinogenesis [41]. To protect the cell from free-radical-mediated damage, living organisms have evolved antioxidant enzymes and substances, e.g., superoxide dismutase (SOD), catalase, glutathione peroxidase (GPX) and glutathione (GSH), to remove excess O_2_^•−^ and H_2_O_2_ [38]. Among ROS, O_2_^•−^ plays a central role in inflammation. O_2_^•−^ is a pro-inflammatory compound that damages e.g., endothelial cells and promotes the migration of neutrophils [42]. The SOD catalyses dismutation of O_2_^•−^ by transforming it into H_2_O_2_ and dioxygen (O_2_). Subsequently, the H_2_O_2_ is eliminated by GPX or catalase. The GSH is the most abundant free thiol in eukaryotic cells which maintains an optimal intracellular redox environment for cellular proteins [43]. It protects cells from oxidative stress by reducing disulfide bonds of cytoplasmic proteins to cysteines. During this process, reduced GSH is oxidized to glutathione disulfide (GSSG). It was shown that GSH regulates redox signaling by altering the level of total GSH and the ratio of its oxidized (GSSG) to reduced (GSH) forms [44]. Aside from GSH, the glutathione system includes glutathione reductase, GPX and glutathione *S*-transferase (GST).

The respiratory burst is an important occurrence in inflammation, characterized by production of O_2_^•−^ and other ROS including H_2_O_2_ and HO^•^ [45]. In all cell types exhibiting respiratory burst, the generation of O_2_^•−^ is dependent on assembly and activation of the NADPH oxidase complex [46]. It has been shown that most cell types, e.g., neutrophils or eosinophils, generate intracellular ROS and the generation of ROS through the activation of the phagocyte NADPH oxidase (NOX) (gp91phox/NOX2) has been considerably studied [47]. Studies on eosinophil NOX activation have shown strong similarities between eosinophils and neutrophils in assembly and activation of the complex. Nevertheless, eosinophils generate up to 10-fold more extracellular O_2_^•−^ than neutrophils, which may be caused by elevated expression of NADPH oxidase in eosinophils [47]. Translocation and assembly of NADPH oxidase are essential for regulated O_2_^•−^ generation in phagocytes. Activation of this complex is dependent on receptor stimulation of intracellular regulatory Rho-related GTPases, especially Rac1 or Rac2 [48]. The contribution of Rac proteins during production of ROS in non-phagocytic cells have been shown in many studies. In a few recent studies a link between Rac signaling and the activation of the NOXes was shown [49]. For example, a Rac-mediated production of ROS was found in HeLa cells and was implicated in IL-1β-mediated activation of nuclear factor κB (NF-κB) [50]. Rac-mediated production of intracellular ROS is concerned in signal transduction, downstream of cytokine receptors [49]. A comprehensive review of NADPH oxidase-mediated redox signaling was given by Jiang and co-workers, elucidating the role of ROS in stress response and tolerance [51]. Thus, ROS are involved in inflammation via two different functions: the killing of bacteria (“respiratory burst”) and mediation of intracellular pro-inflammatory signaling. Furthermore, ROS resulting from the respiratory chain do also regulate cell death associated with mitochondrial dysfunction.

### 2.2. ROS Formation and the Resulting Early Cell Death Induced by TcdB

As mentioned above, induction of non-apoptotic cell death was reported for both TcdA and TcdB. In 2012, Chumbler and co-workers found that TcdB-treated epithelial cells and porcine colonic tissue undergo a rapid, necrotic cell death which is not dependent on autoprocessing and GTD release of the toxin [52]. In TcdB-treated cells the caspase-3/7 activation was not induced although loss of membrane integrity and associated release of LDH and HMGB1 as well as ATP depletion were promoted. It was shown that at low picomolar concentrations the cells undergo the characteristic cell rounding (cytopathic effect), but cells treated within the nanomolar range of TcdB lost their membrane integrity. As clearly shown by the Lacy group in 2012 and 2013 this cytotoxic effect does not require autoproteolytic release of the GTD or full glucosyltransferase activity supporting the hypothesis that glucosylation of the Rho family GTPases might not be involved in the cell death pathway [52,53]. The same observation was made by Donald and co-workers, who showed that even mutated TcdB lacking the glucosyltransferase activity as well as autoproteolytic activity exhibited relevant residual cytotoxicity [54]. The residual cytotoxicity after three days incubation with TcdB in that study was roughly 5000 fold less than wildtype TcdB. This difference between wildtype and glucosyltransferase deficient TcdB was also estimated in a further study by Wohlan and co-workers, who determined EC50 values for acute cytotoxicity for TcdB and a glucosyltransferase deficient mutant of TcdB, incapable of Rho-glucosylation [55]. Both toxins showed similar EC50 values for induction of early cell death, which was about 4000 fold higher than the EC50 for Rac1 glucosylation/cell rounding of wild type TcdB. Wohlan and co-workers also described the morphological changes induced by high concentrations of TcdB, which are dominated by extreme chromatin condensation, cell shrinkage and blistering of the nuclear envelope [55]. Since affected cells showed histone dephosphorylation and completed cell cycle until arrest in binuclear (4n) state, the authors suggested a kind of programmed cell death. Due to intense chromatin condensation being the lead symptom for early cell death, we used the term pyknosis. TcdB and glucosyltransferase deficient TcdB both induced ROS production and the cytotoxic effect can be reduced via apocynin, an inhibitor of the NADPH oxidase. Stronger evidence for the involvement of the NADPH oxidase was given by Farrow and co-workers, who showed that knock down of NOX1 or NOX3, both Rac1-dependent subunits of the NADPH oxidase complex, strongly reduced TcdB-induced cell death. This suggests that the kinetic of Rac1 glucosylation is crucial whether pyknosis can be triggered by TcdB or not. Further, the kinetic of ROS production and Rac1 inhibition might also be critical for pyknotic cell death associated with programmed cell death or necrosis [55]. H_2_O_2_ was described to be associated with pyknotic and caspase-independent cell death [56]. These findings were in line with studies providing evidence that the cell death was independent of RhoA and Cdc42 expression levels, but dependent on the presence of Rac1 [53].

Taken together, these findings suggest a pathway in which Rac1 is involved in the recruitment and assembly of the NADPH oxidase (NOX) complex that is triggered by TcdB at high concentrations. The inverse correlation of Rac1 expression and ROS production indicates activation of a NOX-dependent pathway on toxin stimulation. This suggests that the TcdB cytotoxicity is the result of NOX-mediated ROS production and is a glucosyltransferase-independent process [53,55]. Time course experiments showed that ROS were detectable within 1.5 h post intoxication. LDH release was not detectable until 2.5 h post intoxication. The early production of ROS and the following LDH release clearly establish the sequence of ROS production preceding cell death [53]. The essential role of the NADPH oxidase as well as specific detection of ROS with dihydroethidium clearly indicates that superoxide radicals (O_2_^•−^) rather than H_2_O_2_ are the cause for the cytotoxic effects of TcdB. In combination with cellular depletion of ATP, this is in line with a necrotic, non-apoptotic cell death [57,58]. To date, the precise molecular mechanism by which TcdB induces ROS-dependent cell death has not been clarified. The requirements of high toxin concentrations, efflux of ATP and the lack of mutated TcdB, which does not integrate into the plasma membrane to induce cytotoxic effect, suggest membrane interaction as reason for generation of NADPH-oxidase derived ROS [17,53,59]. This would be in accordance with the mechanism by which TcdA was speculated to induce ROS or suggested to increase cytosolic ROS by release from mitochondria [60,61]. Recent studies also showed that the abundance of either of the two receptors reported for TcdB, *i.e.*, chondroitin sulfate proteoglycan-4 (CSPG4) or poliovirus receptor like protein 3 (PVRL3) is important for the cytotoxic effect [16,17]. Likewise, interference of toxin/receptor interaction by methyl cholate also decreased cytotoxicity of TcdB [10]. These observations suggest three determinants for TcdB-induced increase in ROS: (i) high toxin concentration (nanomolar range); (ii) high receptor abundance and (iii) functional Rac1. However, neither the functional domain of TcdB nor the precise molecular mechanism is known by which ROS-mediated cell death is triggered.

Induction of ROS-dependent cell death seems to be specific for TcdB, since treatment of cells with neither TcdA [53] nor with TcdBF, another toxinotype of TcdB (variant TcdB from strain 1470, Serotype F) [55] resulted in comparable ROS production. This is also in line with the finding that TcdA does not induce rapid necrosis in HeLa cells [52]. From the three premises mentioned above (toxin concentration, receptor abundance, functional Rac1) it can be deduced that a high intracellular toxin concentration has to be achieved for the cytotoxic effect. The different EC50 values as surrogate for intracellular levels of TcdA and TcdB towards most cells might reflect why TcdB but not TcdA induces early cell death. In fact, only one publication reported on cytotoxicity of TcdA at very high concentrations that was independent of the glucosyltransferase activity and might correlate with cytotoxic effect of TcdB [54]. A correlation with ROS production was however not investigated. Nevertheless, in few publications it was reported that TcdA also induces ROS [60,61,62].

### 2.3. ROS Formation Induced by TcdA

An early observation was made 1999 by Qiu and co-workers, who reported that ROS play a role in TcdA-induced enteritis in rats [62]. In that study, ROS were involved in the pathogenesis of experimental colitis in animal models and in idiopathic inflammatory bowel disease of humans. The authors found that TcdA causes a significant increase in hydroxyl radical and hydrogen peroxide production in intestinal microsomes. The ROS production was inhibited by pre-treatment with either DMSO, a ROS scavenger or with superoxide dismutase (SOD), metabolizing superoxide to hydrogen peroxide. The ileal mucosal xanthine oxidase seemed not to be directly involved in TcdA-associated intestinal response considering that ROS primarily originated from neutrophils [62]. It is noteworthy that in this study about 40 nM TcdA was used which was purified from *C. difficile* culture supernatants. However, the purity of isolated native TcdA or TcdB from *C. difficile* cultures is important. Considering that TcdB induces significant ROS-mediated cell death from 0.1 nM on, a purity of TcdA >99% concerning cross contamination with TcdB has to be warranted to be safe from unspecific effects at this concentration range. The involvement of ROS in TcdA-induced intestinal inflammation was also reported in a further study [61]. There, ROS were release in human intestinal epithelial cells within 10 minutes of TcdA exposure. The ROS were supposed to activate the p38 pathway as an immediate-early response which led to induction of cyclooxygenase-2 and synthesis of PGE2 triggering subsequent ileal inflammation and fluid secretion. Additionally, He and co-workers reported that TcdA directly damages isolated CHO cell mitochondria by generation of reactive oxygen species most probably contributing to cytoskeletal damage. TcdA bound to CHO cell membranes were rapidly internalized into the cell, localized to mitochondria, and caused a rapid decrease in ATP concentration within 15 minutes of exposure. Further, TcdA caused a depletion of mitochondria membrane potential and release of ROS. The mitochondrial localization and dysfunction appeared before the onset of cell rounding, indicating that damage of mitochondria by TcdA might also be an early event in cytotoxicity [60]. In another study, however, recombinant TcdA failed to directly affect mitochondria whereas native TcdA from *C. difficile* cultures showed cell type specific effects [63]. Thus, purity of toxin charges and cell types used for investigations might explain different or even controversial results. On the other hand, TcdA-treated Caco-2 cells showed significant increase in abundance of ROS-metabolizing enzymes such as manganese superoxide dismutase (SOD2), catalase, or glutathione S-transferases (GSTA1, GSTA2), indicating increased mitochondrial oxidative processes [4]. These ROS metabolizing activities, however, were detected 24 h post intoxication and are suggested to result from enhanced lipid metabolism which produces lipid peroxides as by-products. The involvement of ROS in the cytotoxic effect of TcdA is more cryptic than it has been shown for cytotoxicity of TcdB.

### 2.4. Toxin-Induced ROS Production in Neutrophils

NADPH oxidase derived ROS do not only regulate cell death/survival, pro-inflammatory signaling or contribute to cell cycle regulation, they are also important in antimicrobial defense when produced in cells of the native immune system [64]. The phagocytic NADPH oxidase complex build around NOX2 is regulated by Rac2 and Rac1 with different shares, depending on cell type (neutrophils, eosinophils, monocytes or macrophages) and species. Recently, Goy and co-workers described a further effect of TcdB: activation of neutrophils by targeting the formyl peptide receptor 1 (FPR-1) [65]. TcdB was able to activate neutrophils as indicated by a rise in intracellular-free Ca^2+^, increased ROS production, integrin activation and degranulation, as it is known from fMLF. This effect was concentration-dependent but was clearly visible only at concentrations of 10 nM or higher. TcdB showed the same profile of rise in intracellular-free Ca^2+^ as the formylated peptide fMLF. Interestingly, a *C*-terminally truncated TcdB1-1852 and also the isolated GTD were even more potent in neutrophil activation than the full-length toxin. Neither full-length TcdA nor TcdA1-1874 were able to activate neutrophils, even when applied at concentration of 300 nM [65]. Desensitation experiments and application of cyclosporin H as specific inhibitor strongly suggest a ligand effect of TcdB on FPR-1. The fact that the isolated *N*-terminal glucosyltransferase domain of TcdB shows a ligand effect on FPR-1 implicates that even a non-cytotoxic fragment of TcdB has pro-inflammatory effects, again with ROS as a central player. Earlier findings also reported about direct effects of TcdA on human granulocytes at high nanomolar concentrations [66]. In that study TcdA induced rise in intracellular free Ca^2+^ but failed to induce generation of superoxide anions.

The first attempts have been made to investigate the role of ROS in toxin-induced damage of the intestine: Farrow and co-workers applied TcdB to porcine colonic explants and verified ROS production in the intestinal mucosa. Antioxidant *N*-acetylcysteine also reduced TcdB-induced tissue damage in human colonic explants [53]. It is, however, hard to distinguish between ROS produced by neutrophils/eosinophils that infiltrated the tissue and those produced by other cells in tissue experiments. Future studies with neutrophil/eosinophil-depleted mice might answer this question.

**Figure 1:**
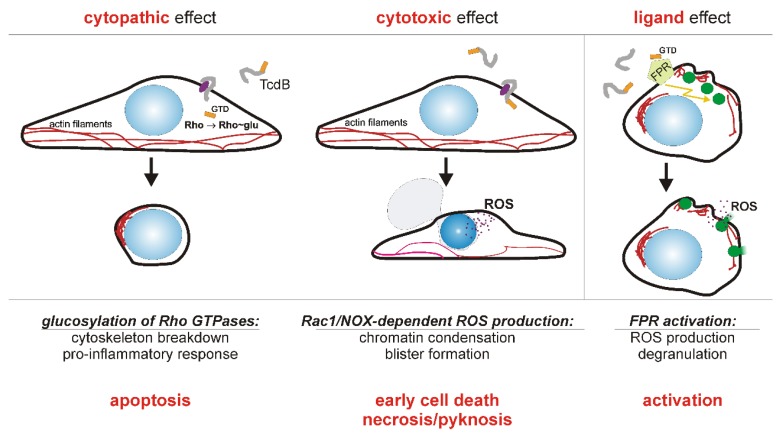
Model of how TcdB affects target cells by three different mechanisms. The cytopathic effect (left panel) results from intracellular glucosylation of the Rho GTPases, by which these signaling proteins are inhibited. Toxin receptor is shown in purple, the *N*-terminal glucosyltransferase domain (GTD) of TcdB is shown in orange. As a consequence of Rho GTPase glucosylation, stress-activated pro-inflammatory signaling takes place and eventually caspase-dependent apoptosis is triggered. The cytotoxic effect (middle panel) is the intracellular induction of an early cell death due to Rac1/NADPH oxidase (NOX) derived reactive oxygen species (ROS). Cell death is associated with chromatin condensation (pyknosis) and blistering. Early cell death is triggered only by high concentrations, more than 1,000 fold higher than the cytopathic concentration. Cells of the hematopoietic linage respond to TcdB at nanomolar concentration with formyl peptide receptor (FPR) signaling (right panel). Even extracellular and thereby non-cytotoxic *N*-terminal fragments of TcdB show ligand effect on FPR-1. Since the cytotoxic effect initially requires functional Rac1 and the cytopathic effect is only displayed by viable cells, it can be assumed that both effects mutually exclude each other.

All data can be summarized in a model where TcdB induces ROS in various cells by two different mechanisms as depicted in Figure 1.

### 2.5. Prevention of ROS-Mediated Cell Damage via Antioxidants

Dimethylsulfoxide, SOD1, pharmacological reagents like *N*-Acetylcysteine (NAC), the Rac1 inhibitor NSC 23766 as well as diphenyleneiodonium (DPI) were all described to decrease ROS level elevated by treatment of cells with TcdB [53,62]. DPI and NSC 23766 have an effect upstream of ROS formation by inhibiting the flavocytochrome enzymatic core of the NOX complex (DPI) or by inhibiting a Rac1 guanine nucleotide exchange factor (NSC 23766). Sun and co-workers reported that cells pre-treated with NAC or DPI showed reduced apoptotic cell death. In that study, NAC or DPI, which attenuate ROS levels, were shown to inhibit apoptosis caused by rTcdB in mouse colonic carcinoma cell line CT26 [67]. The same group, however, also reported on TcdB-induced endoplasmic reticulum stress as reason for non-apoptotic cell death in the same cell line [68]. There, non-apoptotic cell death was induced by a TcdB mutant lacking glucosyltransferase activity. Apparently, as discussed above, wild type TcdB affects cells by glucosyltransferase-dependent and ‑independent synergistic effects. Considering that the ROS mediated cell death is induced by a GT-independent mechanism, antioxidants presumably can completely prevent the cytotoxic effect of TcdB. Farrow and co-workers also tested the protective effect of these compounds on viability of TcdBtreated HeLa cells. The inhibition of ROS generation was sufficient to protect cells from TcdB-induced necrosis [53]. Further, porcine colonic explants were treated with TcdB to investigate whether ROS are produced in the context of colonic tissue and whether DPI or NAC can prevent TcdB-mediated tissue damage. ROS production induced by 10 nM TcdB could be attenuated in tissues pre-treated with DPI. In line with this, NAC, a Food and Drug Administration-approved antioxidant was tested for the human colonic explants. In these explants 100 nM TcdB caused significant damage to the surface epithelial layer which was reduced by pre-treatment with NAC [53]. In cell culture assays, further antioxidant drugs like apocynin or other small molecules were positively tested for their ability to inhibit cytotoxic effect of TcdB [10,55]. The benefit of antioxidant treatment in protection against TcdB further highlights the importance of ROS production in TcdB-induced necrosis [69].

### 2.6. The Role of ROS in C. difficile Infection

The question arises whether the production of ROS by host cells is an advantage or a disadvantage for *C. difficile*. Do ROS play a relevant role in infection process at all, and if so, how does *C. difficile* protects itself against ROS? As stated above, ROS production either intracellular or in terms of respiratory burst is triggered only by relatively high TcdB concentrations within the nanomolar range. It is still questionable whether these concentrations can be locally achieved within the colon. Ryder and co-workers estimated toxin concentration to about 40 pM in stool of patients suffering from *C. difficile* infection in 2010 [70]. A more recent publication also quantified TcdB in human stool specimens and found median concentrations of less than 10 pM [71]. Assumed that the local concentration of TcdB in proximity of adherent clostridia is 30-fold higher than measured in diluted stool, this would represent a concentration sufficient for ROS induction, at least in more susceptible cells.

In general, ROS do have a very short lifetime (10^−9^ to 10^−6^ s) and immediately react with proximate molecules. Thus, extracellular ROS produced by granulocytes supposedly affect microbes stronger than intracellular ROS separated from microbes by a lipid bilayer. Currently the relevance of intracellular ROS in infection process and its role in generation of an oxidative micromilieu has to be considered as second-tier. Nevertheless, anti-oxidative enzymes are important for pathogenic *C. difficile*. The study of Girinathan and co-workers showed that the glutamate dehydrogenase (GDH), which is an important metabolic enzyme, is important for the normal growth of *C. difficile*. The presence of active extracellular GDH may protect *C. difficile* against H_2_O_2_ [72]. The GDH enzyme of *C. difficile* is NAD-specific and mediates the oxidative deamination of glutamate to produce α-ketoglutarate and ammonia [73]. In turn, α-ketoglutarate is important for adjusting oxidative stress in prokaryotes (and eukaryotes). In the study by Girinathan, the gluD mutant is more sensitive to H_2_O_2_ than the parent strains, which suggest that the α-ketoglutarate generated through the action of GDH in *C. difficile* can contribute to H_2_O_2_ tolerance [72]. *C. difficile*-associated diseases are characterised by release of inflammatory cytokines up to intestinal inflammation caused by the host immune response. The production of extracellular GDH may be one possibility for *C. difficile* to defend itself from ROS generated during the host immune response [72]. Interestingly, extracellular GDH is a marker enzyme used for sensitive and quite specific detection of toxigenic *C. difficile* strains, which is not present in atoxigenic strains [74,75]. Additionally, Hillmann and co-workers showed that a deletion of a peroxide repressor (PerR)-homologous protein in *C. acetobutylicum* resulted in a higher resistance to H_2_O_2_ and activities of NADH-dependent scavenging of H*_2_*O_2_ and organic peroxides increased [76]. This peroxide repressor is a member of the Fur protein family and genes encoding proteins of the Fur family were found in all selected clostridia, including *C. difficile*. In the non-stressed *C. acetobutylicum* ΔperR strain, the transcription levels of several members of the putative PerR regulon, which were previously identified to be either induced by H_2_O_2_ or O_2_, were raised. The PerR-like protein acts as repressor of proteins involved in the oxygen defense of *C. acetobutylicum* [76]. Further, the viability of the wild type was severely affected by H_2_O_2_ while even higher concentration of H_2_O_2_ did not drastically reduce the survival of *C. acetobutylicum* ΔperR. The ability to scavenge e.g., H_2_O_2_ or superoxide was also increased in the mutant strain. Further, an approximately threefold increase in SOD activity in the perR-deleted strain was observed [76]. Thus, anaerobic clostridia do have redundant strategies to control scavenging of ROS and to survive oxidative stress.

It is still speculative whether environmental oxidative stress in the colon can be beneficial for pathogenic microorganisms in competition with commensals. In this regard, toxin-induced ROS might even contribute as indirect biocides to reduce commensals and thereby facilitate further colonization of *C. difficile* in the gut. On the other hand, it is known that the commensal microbiota themselves induce ROS in the gut epithelium and that ROS are important mediators for communication with the innate immune system [77]. Moreover, human β-defensin 1, which acts against anaerobic Gram-positive bacteria, is activated by reducing disulfide bridges [78]. In this manner, ROS indirectly act against *C. difficile*.

Tremendous progress has been made in genetic engineering of clostridia/*C. difficile* by developing the ClosTron system or the pyrE-based allelic exchange [79,80]. These techniques now allow investigation of the role of the oxidative stress response genes in *C. difficile* infection and pathogenesis and clearly will help to understand the role of ROS in infection in future.

## 3. Conclusions

Numerous reports document the fact that TcdA, but most of all TcdB, unfolds a cytotoxic effect by ROS. An increase in ROS is triggered by two separate and glucosyltransferase-independent mechanisms. Since ROS induce early cell death, affected cells do show different responses compared to cells suffering from the classical cytopathic effect which are attributed to apoptosis. Thus, ROS-mediated cell death has to be considered an appreciable quality of at least TcdB that contributes to pathogenesis. ROS as pro-inflammatory mediators triggered by TcdA and TcdB are not in contradiction with survival of the anaerobic *C. difficile*, since this microorganism provides efficient systems to control intermediate oxidative stress.
